# Pathogenic features and clinical characteristics of acute community-acquired lower respiratory tract infections

**DOI:** 10.12669/pjms.40.7.7899

**Published:** 2024-08

**Authors:** Na Li, Xixin Yan, Zhiwei Lu, Xiaonan You, Shengfen Yang

**Affiliations:** 1 Na Li, Department of Infectious Diseases, The First Department of Pulmonary and Critical Care Medicine, The Second Hospital of Hebei Medical University, Hebei Key Laboratory of Respiratory Critical Care Medicine, Hebei Institute of Respiratory Diseases, Shijiazhuang 050000, Hebei, China; 2 Xixin Yan, The First Department of Pulmonary and Critical Care Medicine, The Second Hospital of Hebei Medical University, Hebei Key Laboratory of Respiratory Critical Care Medicine, Hebei Institute of Respiratory Diseases, Shijiazhuang 050000, Hebei, China; 3 Zhiwei Lu, Department of Pulmonary and Critical Care Medicine, Baoding No.1 Central Hospital, Baoding 071000, Hebei, China; 4 Xiaonan You, Department of Infectious Diseases, Baoding No.1 Central Hospital, Baoding 071000, Hebei, China; 5 Shengfen Yang, Department of Infectious Diseases, Baoding No.1 Central Hospital, Baoding 071000, Hebei, China

**Keywords:** Acute Community-Acquired Lower Respiratory Tract Infections, Pathogenesis, Clinical Characteristics

## Abstract

**Objective:**

To investigate the pathogen distribution and clinical characteristics of acute community-acquired lower respiratory tract infections (CALRTIs).

**Methods:**

This was a retrospective study. The clinical data of 218 patients with CALRTIs admitted to Baoding No.1 Central Hospital from December 2021 to December 2022 were retrospectively collected and were divided into two groups according to the results of polymerase chain reaction(PCR) testing using a nasopharyngeal swab: streptococcus pneumoniae positive group(observation group) and non-streptococcus pneumoniae positive group(control group). Clinical symptoms, blood gas analysis indicators were compared between the two groups.

**Results:**

Haemophilus influenzae and Staphylococcus aureus, as well as virus and atypical pathogen infection, were the predominant pathogenic bacteria in both groups. No statistically significant differences were observed in the positive rates of sputum smear, sputum culture, respiratory virus detection and atypical pathogen detection between the two groups(P>0.05). However, the control group had a higher detection rate of gram-positive bacteria, gram-negative bacteria and Legionella pneumophila in sputum smears than the observation group, with a statistically significant difference(P<0.05). One death occurred in each group, with no significant difference in mortality and six in each group left the hospital or were transferred due to deterioration, with no significant difference in improved discharge rates.

**Conclusion:**

Acute community-acquired lower respiratory tract infections(CALRTIs) take bacteria, viruses and atypical pathogens as its leading pathogenic bacteria. In the treatment of patients with acute CALRTIs, early pathogenic examination should be performed to assist in guiding antibiotic therapy for rapid control, early recovery and ameliorated clinical outcomes.

## INTRODUCTION

Community-acquired lower respiratory tract infections (CALRTIs) are considered a major cause responsible for mortality among infectious diseases. They are typical clinical diseases with frequent morbidity, among which acute morbidity is common, causing about four million deaths worldwide every year.^1^-^3^ With viruses, bacteria and atypical pathogens as the dominant pathogenic bacteria, CALRTIs are mainly geographically distinctive due to differences in medical conditions, geographical environment and economic conditions.^4^,^5^ If the etiology of CALRTIs can be identified in clinical care, not only does it have an important epidemiological and public health value, but it also allows for precise treatment to be tailored to the etiology to significantly ameliorate the prognosis of patients. Unfortunately, CALRTIs have undergone a gradual shift in the pathogenic spectrum due to a number of factors including the widespread use of antibiotics, the availability of vaccines, the increasing proportion of an aging population, increased complications, climatic changes and the prevalence of respiratory infectious diseases. Exacerbating the situation are the outbreaks and epidemics of multiple emerging and sudden respiratory infectious diseases in recent decades, such as severe acute respiratory syndrome coronavirus (SARS), avian influenza virus HSN1 subtype (H5N1), the Middle East respiratory syndrome coronavirus (MERS-CoV), H1N1 influenza A virus H1N1 subtype(H1N1) and severe acute respiratory syndrome (SARS-CoV-2), especially SARS-CoV-2 that has been plaguing the world for the last three years, all of which have made CALRTIs a huge burden on society.^6^,^7^ For this reason, the pathogen distribution pattern and clinical characteristics of patients with CALRTIs are important indicators to guide the precise treatment of patients, improve their clinical outcomes and reduce mortality. In this study, patients with CALRTIs admitted to our hospital were retrospectively analyzed for their pathogen distribution and clinical characteristics, so as to provide a clinical basis for the timely treatment and prevention and control of these patients.

## METHODS

This was a retrospective study. The clinical data of 218 patients with CALRTIs admitted to Baoding No.1 Central Hospital from December 2021 to December 2022 were retrospectively collected. All the patients were divided into two groups according to the results of polymerase chain reaction (PCR) testing using a nasopharyngeal swab: streptococcus pneumoniae positive group (observation group) and non-streptococcus pneumoniae positive group(control group), with 121 cases in the former group and 97 cases in the latter group.

### Ethical Approval:

The study was approved by the Institutional Ethics Committee of Baoding No.1 Central Hospital (No.: [2022]047; November 03,2022), and written informed consent was obtained from all participants.

### Inclusion criteria:

- Patients who met the diagnostic criteria for CALRTIs and had a confirmed diagnosis of CALRTIs as the primary reason for hospitalization
- Patients aged ≥18 years with onset ≤7 d and admission ≤72 h.
- Patients with complete clinical data.

### Exclusion criteria:

- Patients who refused to sign informed consent.
- Subjects who are participating in clinical trials or intervention studies of other drugs.
- Patients with dysexpression or accompanied by mental illness, congenital heart disease and other infectious diseases; such as cardiopulmonary resuscitation, trauma, postoperation, burn, shock, sunstroke, neuroendocrine neoplasm, extracorporeal circulation, liver cirrhosis, pancreatitis, mesenteric necrosis and catheter infections.
- Patients with incomplete clinical data.

Nasopharyngeal swab and sputum specimens were collected within 72 hour of admission. The sputum was stored at 4°C and immediately transported to the laboratory for treatment within 24 hour. The sputum was diluted and centrifuged to obtain the supernatant and stored at -70°C until cytokine levels were evaluated. Sputum specimens were tested for influenza A, influenza B, parainfluenza, adenovirus, human parapneumovirus, cytomegalovirus, Bocavirus, coronavirus, Streptococcus pneumoniae, Haemophilus influenzae, Staphylococcus aureus, Moraxella catarrhalis and mycoplasma using the Pathogeno Lung-Q respiratory multiple test kit for respiratory pathogens.

While actively treating the primary cause, relative treatment protocols were employed by clinicians according to the patient’s condition, including anti-infection, spasmolytic and asthmatic relief, expelling phlegm and arresting coughing, fluid replenishing and electrolyte balance.

### Observation indicators:

The length of hospital stay, imaging findings, clinical symptoms, patient signs, treatment outcomes, blood indexes, and etiological test results were compared between the two groups. The blood gas indicators included oxygen saturation (SaO_2_), partial pressure of oxygen (PaO_2_) and arterial carbon dioxide pressure (PaCO_2_), while blood albumin and serum inflammatory indicators included white blood cell count(WBC), procalcitonin(PCT), C-reactive protein(CRP) and erythrocyte sedimentation rate(ESR).

### Statistical analysis:

All data in this study were statistically analyzed using SPSS22.0 software, and the measurement data were expressed as (*χ̅*±*S*). Two independent sample *t* test was employed for comparison between the two groups, and the count data were expressed as n (%). The confidence interval is 95%. Besides, c^2^ test was used for comparison between the two groups, with a P<0.05 indicating a statistically significant difference.

## RESULTS

No statistically significant differences were observed in gender, age, the interval between symptom onset and hospitalization, smoking history, history of previous lung diseases (community-acquired pneumonia, tuberculosis, chronic obstructive pulmonary disease, bronchiectasis and asthma, etc.) and other underlying diseases (diabetes, cardiovascular disease and non-lung tumours, etc.), indicating the comparability of differences between the groups (Table-I).

**Table I:** Comparative analysis of general clinical data between the two groups.

Group	n	Gender (cases) Male/Female	Age (years) (*χ̅*±*S*)	Interval between symptom onset and hospitalization (days) (*χ̅*±*S*)	Smoking history (cases) Yes/No	History of lung disease (cases) Yes/No	Other diseases (cases) Yes/No
Observation group	121	71/50	59.43±17.56	4.36±2.20	40/81	25/96	64/57
Control group	97	65/32	58.62±19.84	4.54±2.13	38/59	19/78	43/54
*t/c^2^* value		1.593	0.320	0.583	0.877	0.039	1.580
*P* value		0.207	0.749	0.560	0.349	0.844	0.209

Fifty-four patients in the observation group had a ground-glass opacity on lung CT compared to 30 patients in the control group, with a statistically significant difference(P<0.05); Thirty patients in the observation group had a pleural effusion compared to 36 patients in the control group, with a statistically significant difference(P<0.05). No statistically significant differences were observed in lung involvement between the two groups(P>0.05). Table-II. No statistically significant differences were observed in PaCO2, PaO2 and SaO2 levels between the two groups at admission (P>0.05). Table-III.

**Table-II:** Comparative analysis of imaging findings between the two groups [n(%)].

Group	Ground-glass opacity on lung CT		Pleural effusion		Lateral lung involvement (unilateral/bilateral)	

	Yes	No	Yes	No	Unilateral	Bilateral
Observation group	54 (44.63)	67 (55.37)	30 (24.79)	91 (75.21)	50 (41.32)	35 (36.08)
Control group	30 (30.93)	67 (69.07)	36 (37.11)	61 (62.89)	71 (58.68)	62 (63.92)
c^2^ value	4.267		3.871		0.621	
P value	0.039		0.049		0.431	

**Table-III:** Comparative analysis of blood gas indicators between the two groups (*χ̅*±*S*).

Group	n	PaO_2_ (mmHg)	PaCO_2_ (mmHg)	SaO_2_ (%)
Observation group	121	87.28±30.96	36.61±5.58	95.60±5.57
Control group	97	82.43±24.00	35.70±5.96	95.87±1.91
*t value*		1.046	0.949	0.384
*P value*		0.297	0.344	0.301

No statistically significant differences were observed in the comparison of WBC, PCT and ESR between the two groups (P>0.05). The albumin level in the observation group was higher than that of the control group, while the CRP level was lower than that of the control group, with a statistically significant difference. (P<0.05).

At admission, the fever in the control group was 80.41%, higher than that of 62.81% in the observation group, and the average body temperature of patients in the control group were higher than that in the observation group, with a statistically significant difference (P<0.05). There was no significant difference in the number of patients with chills, cough, dyspnoea and chest pain between the two groups, with no statistically significant difference (P>0.05). Table-V.

**Table-IV:** Comparative analysis of albumin and inflammatory indexes between the two groups (*χ̅*±*S*).

Group	n	WBC (×10^9^/L)	PCT (μg/L)	CRP (g/L)	ESR (mm/h)	Albumin (g/L)
Observation group	121	9.44±5.13	1.96±9.34	72.34±75.14	52.77±36.62	38.17±6.30
Control group	97	9.84±4.97	2.94±11.47	93.85±82.56	57.60±34.63	35.78±7.27
*t value*		0.573	0.681	1.995	0.980	2.795
*P value*		0.567	0.496	0.047	0.328	0.006

**Table-V:** Comparative analysis of symptoms and signs between the two groups.

Group	n	Body temperature (°C)	Fever (Yes/No)	Chills (Yes/No)	Cough (Yes/No)	Dyspnoea (Yes/No)	Chest pain (Yes/No)
Observation group	121	37.63±1.22	76/45	16/105	99/22	52/69	28/93
Control group	97	38.28±1.19	78/19	13/84	76/21	47/50	23/74
*t value*		3.974	8.044	0.001	0.409	0.652	0.010
*P value*		0.000	0.005	0.969	0.523	0.19	0.921

Prior to admission, antibiotics were partially used in both groups. In the observation group it was 39.67%, which was lower than that of 58.76% in the control group, with a statistically significant difference(P<0.05). One death occurred in each group, with no significant difference in mortality (P>0.05). Moreover, there was no significant difference between the two groups in terms of the proportion of patients on oxygen uptake, invasive ventilation and non-invasive ventilation, with no statistically significant difference (P>0.05). There was neither a statistically significant difference between the two groups in terms of CURB65 scores (P>0.05) nor in terms of rates of discharge in good condition (P>0.05). Table-VI.

**Table-VI:** Comparative analysis of treatment and regression between the two groups.

Group	n	Length of hospital stay (d)	Antibiotic application prior to admission (Yes/No)	Oxygen uptake (Yes/No)	Invasive ventilation (Yes/No)	Non-invasive ventilation (Yes/No)	CURB65 score (points)	Discharged in good condition (Yes/No)
Observation group	121	9.75±4.26	48/73	70/51	0/121	0/121	0.81±0.79	114/7
Control group	97	11.62±5.54	57/40	66/31	1/96	1/96	0.82±0.92	90/7
*t value*		2.811	7.862	2.382	1.253	1.253	0.128	0.184
*P value*		0.005	0.005	0.123	0.263	0.263	0.898	0.668

Complications occurred in both groups during the treatment. The incidence of complications in the control group was 64.95% (63 cases), higher than 45.45% (55 cases) in the observation group, with a statistically significant difference (P<0.05). Comorbidities between the two groups included electrolyte disturbances, hypoproteinemia, anaemia, liver insufficiency and respiratory failure, with a higher proportion of electrolyte disturbances, anaemia, liver insufficiency and respiratory failure in the control group than in the observation group, with statistically significant differences (P<0.05). Table-VII.

**Table-VII:** Comparative analysis of the occurrence of complications between the two groups.

Group	n	Electrolyte disturbance (Yes/No)	Hypoproteinemia (Yes/No)	Anaemia (Yes/No)	Liver insufficiency (Yes/No)	Respiratory failure (Yes/No)	Pulmonary embolism (Y/N)	Lung abscess (Yes/No)	Empyema (Yes/No)	Venous thrombosis (Yes/No)
Observation group	121	24/97	22/99	10/111	8/113	7/114	1/120	5/116	2/119	8/113
Control group	97	34/63	27/70	18/79	15/82	14/83	4/93	3/94	2/95	2/95
*t value*		6.384	2.879	5.095	4.471	4.625	2.612	0.165	0.050	2.546
*P value*		0.012	0.090	0.024	0.034	0.032	0.106	0.685	0.823	0.111

No statistically significant differences were observed in the positive rates of sputum smear, sputum culture, respiratory virus detection and atypical pathogens detection between the two groups (P>0.05). The detection rates of Gram-positive, Gram-negative and Legionella pneumophila on sputum smear were higher in the control group than in the observation group, with statistically significant differences (P<0.05). Tables-VIII and IX.

**Table-VIII:** Comparative analysis of sputum smear and sputum culture results between the two groups.

Group	Sputum smear		Sputum culture		Respiratory virus detection		Atypical pathogen detection	

	Positive	Negative	Positive	Negative	Positive	Negative	Positive	Negative
Observation group	73	21	6	86	30	45	23	60
Control group	50	15	3	63	19	34	12	34
t/c^2^ value	0.012		0.279		0.226		0.039	
P value	0.913		0.597		0.634		0.843	

**Table-IX:** Comparative analysis of sputum culture and pathogen detection rates between the two groups [n(%)].

Pathogens	Observation group (n=121)	Control group (n=97)	χ^2^ value	P value
Sputum culture				
Gram-positive bacteria	59 (48.76)	68 (70.10)	10.085	0.001
Gram-negative bacteria	35 (28.93)	45 (46.39)	7.070	0.008
Influenza virus				
Influenza A virus	12 (9.92)	14 (14.43)	1.045	0.307
Influenza B virus	24 (19.83)	15 (15.46)	0.700	0.403
Atypical pathogens				
Mycoplasma	16 (13.22)	9 (9.28)	0.825	0.364
Chlamydia	2 (1.65)	1 (1.03)	0.153	0.695
Legionella pneumophila	12 (9.92)	3 (3.09)	3.914	0.048

In both groups, the other main pathogens were Haemophilus influenzae, among which there were 14 cases in the observation group and eight cases in the control group, accounting for the first place in other etiology, followed by Staphylococcus aureus. One patient in the observation group also had human metapneumovirus infection, compared to three in the control group, and cytomegalovirus was detected in three patients in the control group, with no statistically significant difference regarding the distribution of other pathogens in the two groups (P>0.05). Table-X.

**Table-X:** Comparative analysis of the distribution of other pathogens between the two groups [n(%)].

Pathogens	Observation group (n=121)	Control group (n=97)	χ^2^ value	P value
Bacteria			0.863	0.353
Staphylococcus aureus	5 (4.13)	3 (3.09)		
Haemophilus influenzae	14 (11.57)	8 (8.25)		
Viruses			1.868	0.172
Human metapneumovirus	1 (0.83)	3 (3.09)		
Cytomegalovirus	0 (0.00)	3 (3.09)		
Bocavirus	2 (1.65)	0 (0.00)		

## DISCUSSION

In the present study, bacteria still dominated acute CALRTIs. Streptococcus pneumoniae was detected in 121 cases (55.50%) of 218 patients, followed by Haemophilus influenzae and Staphylococcus aureus, with viruses and atypical pathogens detected in a small number of patients, which was similar to the results of clinical studies in recent years.^8^,^9^ In the last decade or so, viral CALRTIs have become more frequent and outbreaks of various viral pneumonia epidemics have placed a heavy economic burden on society. In particular, the SARS-CoV-2 outbreak epidemic of the last three years has shown no signs of stopping to date.^10^ To this end, attention should be focused not only on bacterial CALRTIs, but also on viral CALRTIs, and a comprehensive pathogen detection tool should be established to detect and prevent and control new and emergent infectious diseases.

Acute community-acquired lower respiratory tract infections(CALRTIs) are a common disease in clinic with a broad spectrum of pathogens that vary across geographic regions, populations and seasons. Two of the most common pathogens are bacteria and viruses, while mycoplasma, chlamydia and fungi are also highly pathogenic. Therefore, identification of the pathogen in the event of an acute CALRTI is the basis for guiding clinical application of anti-infective drugs.^11^-^13^ However, the wide application of antibiotics and advances in detection techniques have also contributed to the current changes in the pathogenic spectrum of acute CALRTIs.^14^ Previous reports have identified bacteria as the main pathogens of acute CALRTIs, among which Streptococcus pneumoniae and Haemophilus influenzae were most common. In recent years, respiratory viruses, and atypical pathogens such as mycoplasma and chlamydia have taken on an important position.^15^-^17^ Therefore, it is of vital importance to improve clinical cure rates by clarifying the pathogenic distribution of acute CALRTIs.

CT has gradually replaced X-ray examination because of its high resolution. CALRTIs are essentially inflammatory exudation of lung tissue, which, if left untreated, can accumulate as a pleural effusion. In terms of CT imaging, CALRTIs are ground-glass opacity density foci, which may be their early imaging manifestations.^18^ However, the focal distribution and density of patients are varied and their imaging findings are dynamic, taking into account individual patient differences. It was shown in this study that the main imaging findings of CALRTIs were ground-glass opacity (38.53%) and pleural effusion (30.28%). In the treatment of CALRTIs, the use of antibiotics is the preferred therapeutic modality. Antibiotics with good efficacy and safety are the key to treatment, and in clinical practice antibiotics with good sensitivity are usually selected based on sputum cultures and drug sensitivity tests. However, improper antibiotics can easily lead to poor treatment and drug resistance. In this study, most patients in both groups had fever on admission, which was consistent with relevant studies.^19^,^20^ Prior to admission, some patients in the two groups used antibiotics on their own. in the observation group being 39.67%, which was lower than that of 58.76% in the control group(P<0.05). However, the fever in the control group was 80.41%, higher than 62.81% in the observation group, and the average body temperature of patients in the control group was higher than that in the control group(P<0.05). CALRTIs can be quickly controlled if treated promptly and with appropriate antibiotic choices.^21^ According to this study, there was one death in each of the two groups after treatment and most patients were discharged in good condition, with no statistically significant difference (P<0.05).

### Limitations of this study:

It includes the small sample size and the lack of follow-up are two major limitations of our study. In addition, we only analyzed and discussed the cases included in our hospital, which may not be representative enough. We look forward to a multi-center study in the future to reach more comprehensive conclusions.

## CONCLUSIONS

Bacteria, viruses and atypical pathogens remain the main pathogenic factors of acute CALRTIs. While treating CALRTIs patients with conventional treatment regimens, sputum culture, respiratory etiological examination and drug sensitivity test should also be performed as soon as possible for early and timely treatment with sensitive antibiotics, so as to achieve rapid disease control, early recovery and ameliorated clinical outcomes.

### Authors’ Contributions:

**NL** and **XY** carried out the studies, participated in collecting data, drafted the manuscript, are responsible and accountable for the accuracy or integrity of the work. **ZL** and **XY** performed the statistical analysis and participated in its design. **SY** participated in acquisition, analysis, or interpretation of data and drafting the manuscript. All authors read and approved the final manuscript.
